# Tuning Conductance in BODIPY-Based Single-Molecule Junctions

**DOI:** 10.1021/acs.nanolett.5c03764

**Published:** 2025-08-26

**Authors:** Emma York, Ilana Stone, Wanzhuo Shi, Xavier Roy, Latha Venkataraman

**Affiliations:** † Department of Chemistry, 5798 Columbia University, New York, New York 10027, United States; ‡ 148492 Institute of Science and Technology Austria, 3400 Klosterneuburg, Austria; § Department of Applied Physics and Applied Mathematics, Columbia University, New York, New York 10027, United States

**Keywords:** Single-molecule electronics, BODIPY, tuning conductance, density functional theory, self-energy corrections

## Abstract

Here, we present a foundational investigation of charge transport through three BODIPY-based molecules using the scanning tunneling microscope–break junction (STM-BJ) technique. We demonstrate that molecular conductance through the BODIPY core can be measured by introducing aurophilic linkers at the 2,6-positions. By varying these linkers, we systematically modulate the frontier molecular orbital energies and fine-tune transport behavior. Our experimental results are supported by DFT-based calculations, which feature a new computationally efficient correction to standard PBE-level transmission predictions. Together, these findings establish the viability of BODIPY-based systems for molecular junction applications and lay the groundwork for future studies of their single-molecule optoelectronic properties.

## Graphical abstract

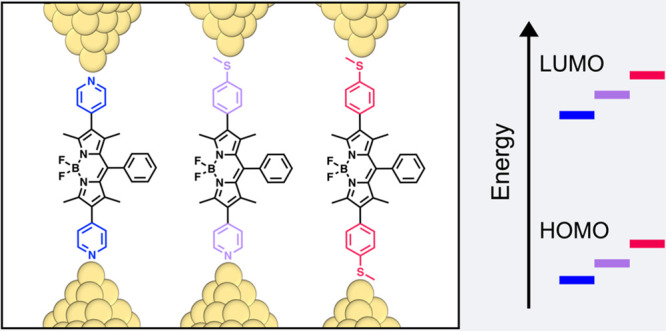

Metal–molecule–metal junctions are valuable platforms for probing relationships between chemical structure and charge transport at the nanoscale.^1–4^ These works expand the molecular design toolbox needed to develop next-generation nanoelectronic devices, where single molecules function as active circuit elements.^5–8^ Recently, these nanoscale devices have garnered interest for their potential use as light-emitting diodes (LEDs).^9–14^ Developing strategies to modulate energy level alignment will be critical in systems where molecular luminescence is harnessed. Difluoroboron-dipyrromethene (BODIPY)-based luminophores offer great potential for such applications but have not been well-studied at the single-molecule level.^15^ BODIPY dyes are particularly attractive scaffolds for single-molecule optoelectronics due to their robust photophysical properties, including strong photoluminescence from the S_1_–S_0_ (π*−π) transition,^16–18^ which can be tuned via modification of the BODIPY core.^16,19–21^ Although BODIPY derivatives have been studied for applications such as solar cells,^22^ OLEDs,^23,24^ photosensitizers,^25^ and sensors,^26,27^ their electronic behavior in single-molecule junctions remains relatively unexplored.^15^ Here, we characterize and tune the electronic properties of junctions formed with three BODIPY derivatives as a foundational step toward future BODIPY-based nanoscale electronic components.

We measure a series of BODIPY molecules terminated with aurophilic pyridyl and thiomethyl linkers at the 2,6-positions on the BODIPY core using the scanning tunneling microscope-based break-junction (STM-BJ) technique and Au-metal electrodes.^28,29^ Pyridine and thiomethyl groups are common linkers that form transient dative bonds to Au electrodes via lone pairs on N and S atoms, respectively.^2^ Transport across a single-molecule junction is largely determined by the energetic alignment between the molecule’s frontier orbitals and the junction Fermi energy (*E*_F_).^30,31^ In pyridine-terminated structures, the LUMO (lowest unoccupied molecular orbital) is stabilized by the presence of electron-withdrawing groups shifting the transmission function toward a LUMO-dominated electron transport regime.^6^ Conversely, thiomethyl groups are electron-donating, raising the HOMO (highest occupied molecular orbital) energy and favoring a HOMO-dominated hole transport regime.^2^ Measuring both symmetrically and asymmetrically anchored systems, we probe the effects of linker substitution on junction transport. We compare our experimental results with theoretical calculations. We first carry out standard DFT-based transmission calculations using a PBE functional. Since these overestimate transmission near *E*_F_,^32,33^ we develop a computationally efficient general correction method to enable comparing the transmission across the series with higher accuracy. Together, our experiments and calculations demonstrate that the BODIPY core serves as a molecular platform where conductance can be tuned by altering the linker.

Figure 1 illustrates our synthetic scheme for incorporating substituents at the 2,6-positions of 1,3,5,7-tetramethyl-8-phenyl-4,4-difluoroboradiazaindacene. Substitution at these positions enforces an orthogonal arrangement of the BODIPY core relative to the junction axis (Figure 1c). Homosubstituted BODIPY derivatives **B1** with thioanisole linkers and **B3** with pyridine linkers are synthesized by first iodinating the 2,6-positions of the BODIPY core, followed by a Suzuki–Miyaura cross-coupling reaction adapted from previous methods^20,34,35^ to introduce either thioanisole or pyridine linkers (Figure 1a). Heterosubstituted derivative **B2** with one thioanisole and one pyridine linker was obtained via a stepwise iodination and bromination at the 2,6-positions, enabling sequential and chemoselective Suzuki couplings with two distinct linker fragments (Figure 1b). Complete synthetic details can be found in the .

**Figure 1:**
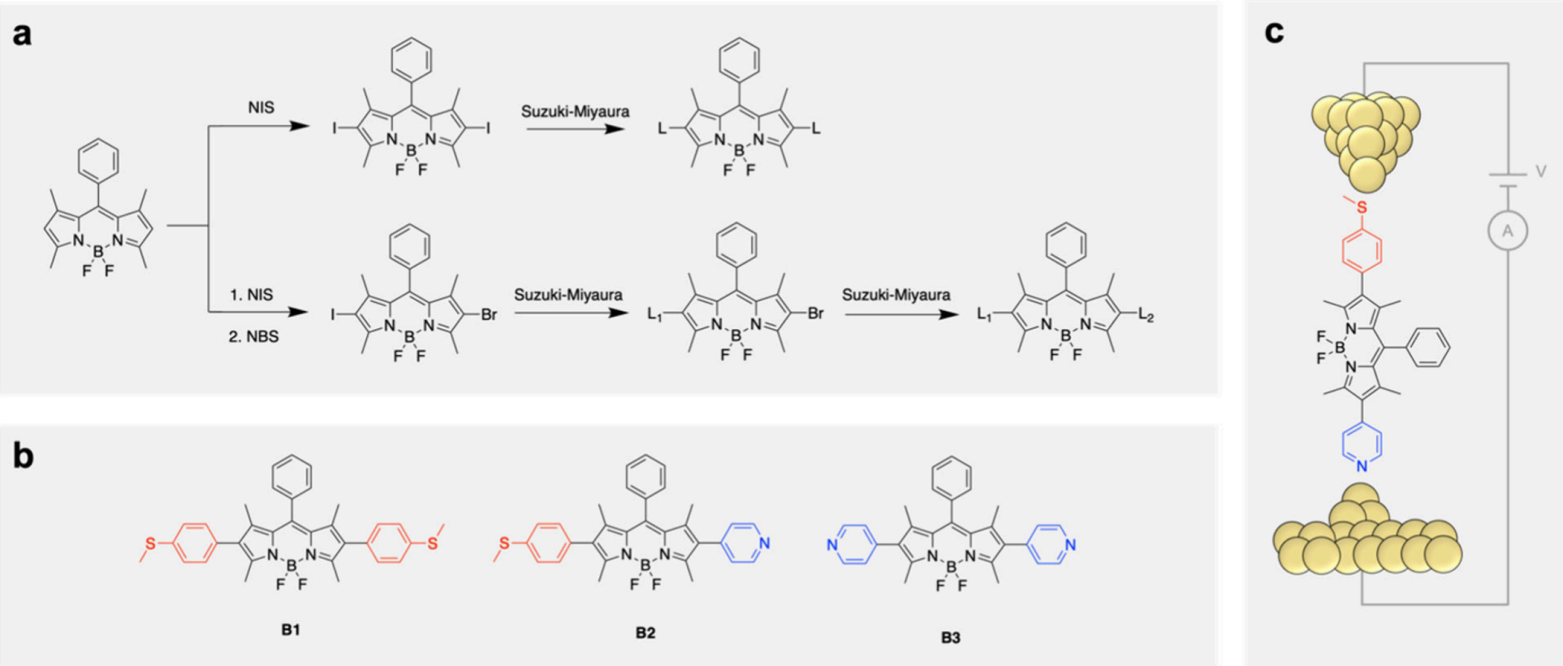
(a) Synthetic scheme for the formation of homosubstituted (top) and heterosubstituted (bottom) BODIPY derivatives **B1**–**B3**. Full experimental details are provided in the . (b) Molecular structures of BODIPY derivatives **B1**–**B3**. (c) Illustration of a single-molecule junction formed using **B2**.

Molecular conductance was measured using an STM-BJ setup that has been described in other works.^29^ A gold tip is repeatedly brought into and out of contact with a gold substrate under an applied bias voltage, while current across the junction is measured to determine conductance (*G* = *I*/*V*). As the tip is withdrawn, a conductance plateau appears close to the quantum of conductance (1*G*_0_ = 2e^2^/*h*), corresponding to a single-atom Au–Au contact. Upon breaking this contact, the conductance typically drops; however, if a molecule bridges the junction (Figure 1c), a second plateau appears at a lower conductance value (<*G*_0_).

Conductance measurements of the BODIPY molecules were carried out using 100 μM solutions of **B1**, **B2**, and **B3** in 1,2,4-trichlorobenzene (TCB) under an applied bias of 500 mV (Figure 2). For each compound, 5000 consecutive conductance traces were recorded and compiled into one-dimensional (1D) and two-dimensional (2D) histograms without data selection. Each molecule displays a clear peak in the 1D conductance histogram (Figure 2a), which was fit to a Gaussian curve to extract the most probable conductance value. Conductance increases across the series in the order **B3** < **B2** < **B1**, with values of 1.5 × 10^–5^ *G*_0_, 2.5 × 10^–5^ *G*_0_, and 5.4 × 10^–4^ *G*_0_, respectively. 2D conductance-displacement histograms (Figures 2b, 2c, and 2d) show conductance plateaus ranging between 10^–4^ and 10^–5^ *G*_0_, with the three compounds exhibiting similar plateau lengths that are typically over a nanometer long.

**Figure 2:**
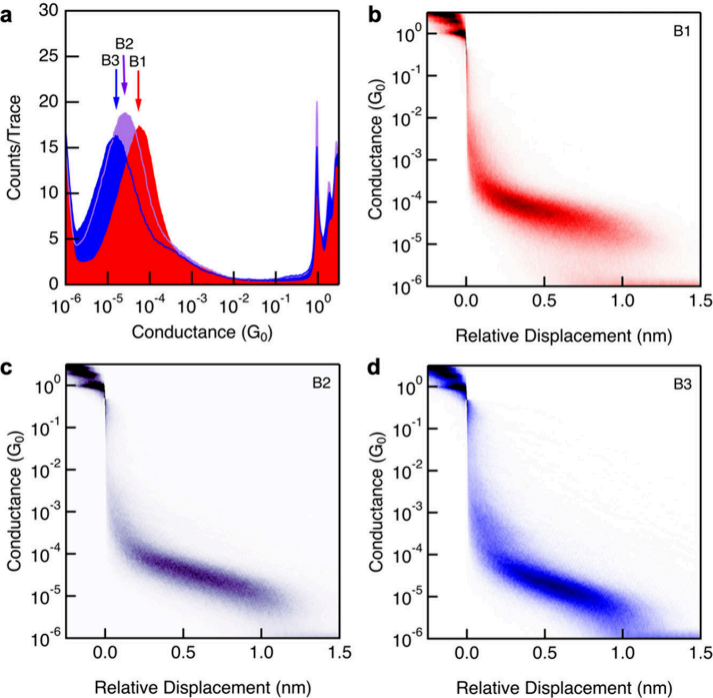
(a) Logarithmically binned one-dimensional (1D) histograms of **B1** (red), **B2** (violet), and **B3** (blue) at +500 mV in TCB generated from 5000 conductance-displacement traces. (b–d) 2D conductance–displacement histograms generated by aligning and overlaying traces from 1D histograms for **B1** (panel (b)), **B2** (panel (c)), and **B3** (panel (d)).

We next probed the bias dependences of the molecular junction currents. Unlike standard conductance measurements, where the tip is withdrawn at a constant rate, in these experiments, the tip is held at the displacement where a junction is expected to form while the bias is ramped up and down. Due to the lower probability of picking up a molecule at the start of the bias ramp and the reduced stability of junctions at high biases, tens of thousands of traces must be collected, and only the traces showing clear signatures of molecular junctions are compiled into 2D current versus time histograms. Figure 3 shows current versus time histograms for BODIPY junctions in which the bias was swept from 0 to 1.5 V and back to 0 V over a 50 ms window. Among the three molecules, the **B1** junction exhibits the greatest bias dependence, with currents exceeding 10^–7^ A at 1.5 V. Additionally, a notable number of ruptured junctions can be observed in the **B1** histogram upon reaching the maximum bias indicating that we are approaching resonant transport. By contrast, **B3** junctions show minimal bias dependence and lower maximum currents (∼10^–8^ A). The asymmetric **B2** molecule shows bias dependence and maximum current between those of the homosubstituted molecules **B1** and **B3**.

**Figure 3:**
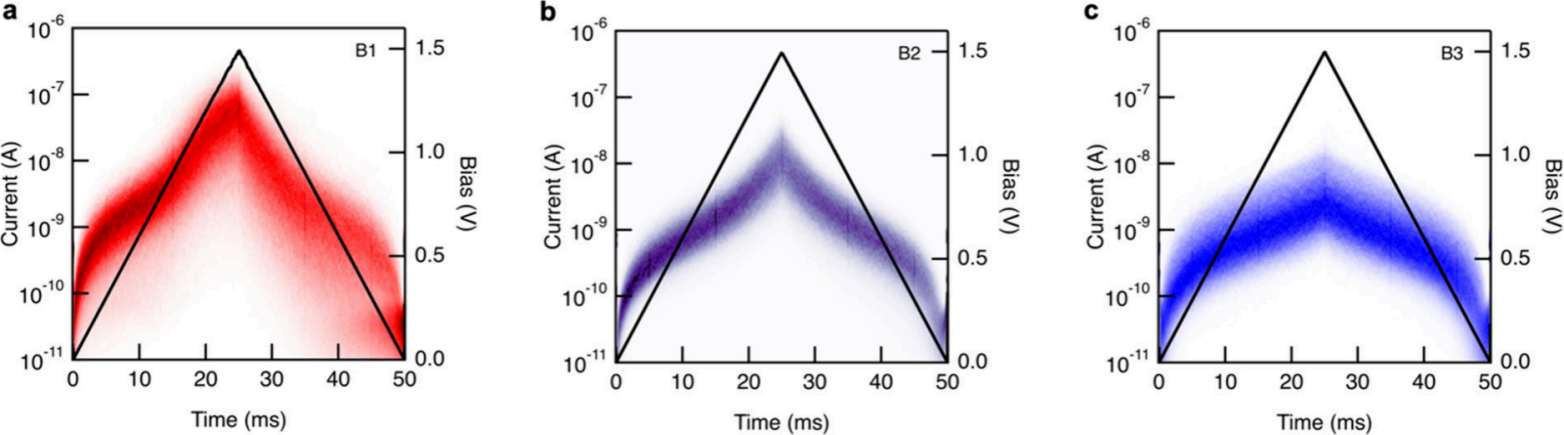
2D current versus time histograms measured while linearly ramping the bias voltage across the junction from 0 V to +1.5 V and back to 0 V over a 50 ms period (black trace). Histograms were compiled from (a) 6814 **B1** junctions, (b) 7260 **B2** junctions, and (c) 6687 **B3** junctions.

We can interpret the observed trend in bias dependence in the context of junction electronic structure by turning to transmission calculations. Transmission functions were first calculated using the nonequilibrium Green’s function (NEGF) formalism as implemented in FHI-aims and the AITRANSS transport package, using the PBE functional.^32,33,36,37^ Calculations were performed for optimized molecular structures connected to 58-atom gold electrodes as detailed in the . It has been noted in prior studies that certain errors are inherent to this method,^32,33^ such as the tendency to underestimate the HOMO–LUMO gap, leading to inaccuracies in the predicted transport properties. Although using a hybrid functional such as B3LYP would partly mitigate this issue in part, such calculations for large systems involving extended gold electrodes are computationally demanding. Alternative methods to correct for this error include using the DFT+Sigma method developed by the groups of Neaton and co-workers^38–40^ or using the self-consistent GW method.^41–43^ However, these are computationally very challenging.

To address these discrepancies, we developed a simpler method to emulate the effects of a B3LYP-level calculation by correcting the PBE-derived transmission functions using calculated orbital energy shifts. We fit each resonance in the calculated PBE transmission function that contributes to the low-bias transmission at an energy *E* with

$$t_{i}\left(E\right)=e^{{i\theta }_{i}}\frac{\Gamma_{i}}{E-\varepsilon_{i}+i\Gamma_{i}}$$

Here, *i* denotes the orbital or resonance number, θ_*i*_ is the phase shift that arises when electron waves traverse the junction across the *i*th orbital, Γ_*i*_ is the coupling of the *i*th orbital to the leads and ε_*i*_ is the energetic position of the orbital relative to the Fermi energy. The total transmission is the sum of all contributions with *T*(*E*) = |∑_*i*_ *t*_*i*_(*E*)|^2^.^44^ We consider eight frontier molecular orbitals (four above and four below *E*_F_) and fit the PBE-based DFT transmission to obtain three parameters (θ_*i*_, ε_*i*_, Γ_*i*_) for each of the eight orbitals. Since the primary error in the PBE-functional-based transmission calculation is the position of each resonance relative to *E*_F_, we determine the energy by which each resonance position ε_*i*_ must be corrected to more accurately capture the transmission.

Toward this goal, we consider the isosurface plot of the scattering states obtained at the transmission resonances and determine the corresponding orbitals in the isolated molecule. For example, Figures 4a and 4b show the isolated molecular HOMO and LUMO along with the scattering states at the two resonances closest to the Fermi energy. The similarity in orbital shape and nodal structure confirms that the transmission resonances immediately below and above *E*_F_ originate from the HOMO and LUMO, respectively. This indicates that we can alter the position of these resonances (ε_*i*_) to obtain transmission functions that are more representative of the experiments. We then calculate orbital energies for an isolated molecule using both the PBE functional and the B3LYP functional. We determine the energy difference between these two calculations as Δε = ε_B3LYP_ – ε_PBE_. This correction does not include errors that arise due to the polarization correction.^38,39^ However, since these BODIPY molecules are long (the Au–Au separation in the junctions is ∼1.9 nm), the polarization correction is small, compared to the self-energy correction. Note that Δε is negative for occupied orbitals and positive for unoccupied orbitals. We then recompute the total transmission to obtain

$$T_{\mathrm{corr}}\left(E\right)={\left|\underset{i}{\sum }e^{{i\theta }_{i}}\frac{\Gamma_{i}}{E-\left(\varepsilon_{i}+\Delta \varepsilon \right)+i\Gamma_{i}}\right|}^{2}$$

Note that we restrict our model to one that has the same coupling at both electrodes. Although this could cause an issue for **B2**, which is asymmetrically linked, the LUMO orbital which is closest to the Fermi energy has a transmission close to 1, indicating that it is equally coupled to the two sides. The Δε values are tabulated for each molecular junction in the . Figure 4c shows a fit of the PBE-derived transmission, and the final approximation using B3LYP-derived transmission resonance corrected following the equation above for the **B2** system (similar figures for **B1** and **B3** are shown in the ). This provides a computationally efficient way to improve the accuracy of the transmission function and allows for a more meaningful comparison with experimental data.

**Figure 4:**
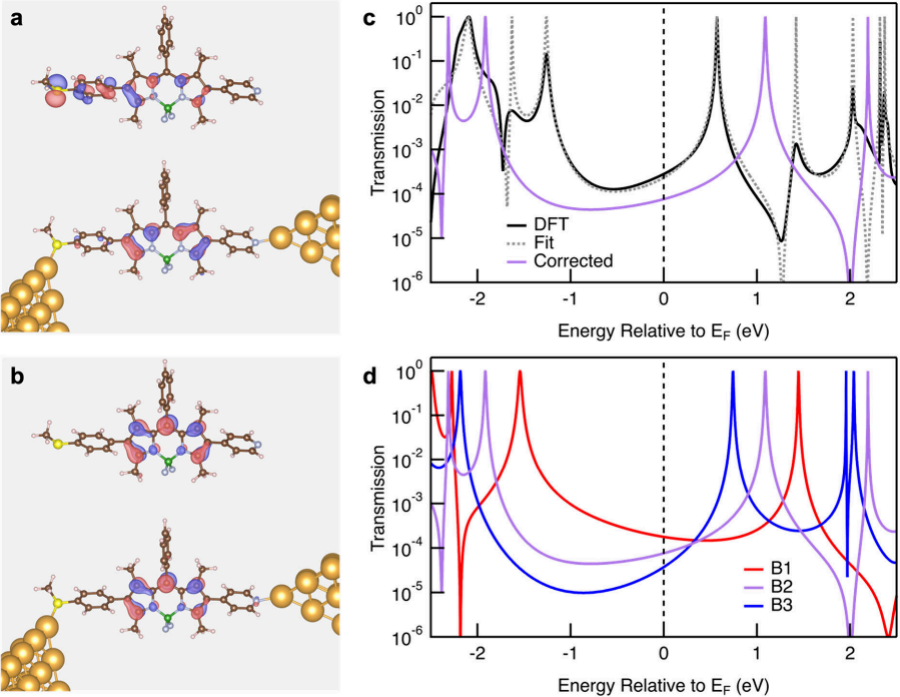
(a) HOMO and (b) LUMO obtained from DFT calculations for an isolated **B2** molecule relaxed with a PBE functional, and the scattering states at the two resonances closest to *E*_F_ (at −1.260 and 0.571 eV) in the PBE-derived transmission calculation with 58-atom Au electrodes. (c) Sample transmission function showing the fit and corrected transmission for a **B2** junction. (d) Approximated transmission functions for **B1, B2**, and **B3** junctions.

We apply this same procedure to obtain corrected transmission functions for the other two junctions. The results are shown in Figure 4d. Near *E*_F_, the conductance values derived from our corrected transmission functions follow the trend **B3** < **B2** < **B1**, in agreement with experimental results. By contrast, the PBE based transmissions overestimate the conductance significantly and show trends that are not in alignment with the experiment (see the for details). We see from the corrected transmissions that the HOMO–LUMO gap remains consistent across all three systems, indicating that our substitution at the 2,6-positions of the BODIPY core does not significantly perturb the intrinsic ground-state electronic structure. We hypothesize this is due to the twisting of the linker aryl groups which disrupt planarity and electronically decouple the BODIPY core from the electrodes. Instead, the HOMO and LUMO shift together incrementally with linker identity, demonstrating that chemical modifications at these positions offer a controlled strategy for fine-tuning orbital alignment within the junction.

In summary, we investigate single-molecule charge transport through BODIPY derivatives functionalized with pyridine and thioanisole linkers. STM-BJ experiments reveal an increase in conductance across the series **B3** < **B2** < **B1**, consistent with trends in bias dependence and corrected DFT-based transmission calculations. Despite changes in linker identity, the HOMO–LUMO gap remains constant across the series of molecules, indicating that substitution at the 2,6-positions tunes frontier orbital alignment while preserving the intrinsic properties of the BODIPY core. These findings provide a foundation for designing BODIPY-based molecular junctions that combine tunable charge transport where the alignment of transmission resonances can be altered through the use of chemical linkers.

## Supplementary Material









## Notes

Synthetic details, measurement and theoretical procedures, and additional data (PDF)
3D structural depiction of the single-molecule junction formed using **B1** (XYZ)
3D structural depiction of the single-molecule junction formed using **B2** (XYZ)
3D structural depiction of the single-molecule junction formed using **B3** (XYZ)
