# Supplementary material for: Tuning Conductance in BODIPY-Based Single-Molecule Junctions
Source: Nano Lett. 2025 Aug 26;25(36):13697–702. doi: 10.1021/acs.nanolett.5c03764 (PMC12426929; doi:10.1021/acs.nanolett.5c03764)
Supplement: Supplementary file 1 [file nl5c03764_si_001.pdf]

Supporting Information for

Tuning Conductance in BODIPY-based Single Molecule Junctions

Emma York[1,2], Ilana Stone[1], Wanzhuo Shi[1,2], Xavier Roy[1], Latha Venkataraman[1,2,3]

[1]Department of Chemistry, Columbia University, New York, New York 10027, United States

[2]Institute of Science and Technology Austria, 3400 Klosterneuburg, Austria

[3]Department of Applied Physics and Applied Mathematics, Columbia University, New York, New York 10027, United States

Contents:

1. **Synthetic Details**
2. **Conductance Measurements (STM-BJ)**
3. **Density Functional Theory Calculations**
4. **NMR Spectra**
5. **References**

1. Synthetic Details

All commercial reagents and solvents were used as received without further purification. The starting material, 8-phenyl-1,3,7,9-tetramethyl-BODIPY, was purchased from BLDpharm. Reactions were performed under a nitrogen atmosphere using standard Schlenk techniques unless otherwise noted. Final products were dried under vacuum prior to yield determination.

^{1}H NMR and ^{13}C NMR spectra were recorded in $CDCl_3$ on Bruker Avance III (300 and 500 MHz) or Avance IV NEO (800 MHz) spectrometers, as indicated. Chemical shifts (δ) for ^{1}H are reported in ppm relative to tetramethylsilane (TMS), with residual $CHCl_3$ used as the reference (δ 7.26 ppm). For ^{13}C, shifts are reported in ppm relative to TMS, with $CDCl_3$ serving as the reference (δ 77.0 ppm). NMR data are given as chemical shift, multiplicity (s = singlet, d = doublet, t = triplet, m = multiplet), coupling constants (J) in Hz, and integration.

HRMS sample preparation, analysis, and data evaluation were performed by Aikaterina Paraskevopoulou, Mass Spec Service, LSF, ISTA. Samples were diluted in a mixture of ACN:H_2O (70:30 v/v), containing 0.1%FA, at a final concentration of 10 ug/mL. All solvents used were of LCMS grade. The analysis of target analytes was performed by direct infusion using a Q Exactive HF mass spectrometer (Thermo Fisher Scientific, USA), equipped with a heated electrospray ionization (HESI) source and coupled to an Ultimate 3000 UHPLC chromatograph (Thermo Fisher Scientific, USA). The chromatographic column used was an ACQUITY UPLC BEH C18 column (1.7 µm, 2.1 × 50mm; Waters, USA). Xcalibur software 4.3.73.11 was used to control the MS parameters. Data analysis was performed using FreeStyle software 1.8.51.0.

Synthetic details are provided below, with final products abbreviated **B1-B3** and intermediates abbreviated **I1-I4**.

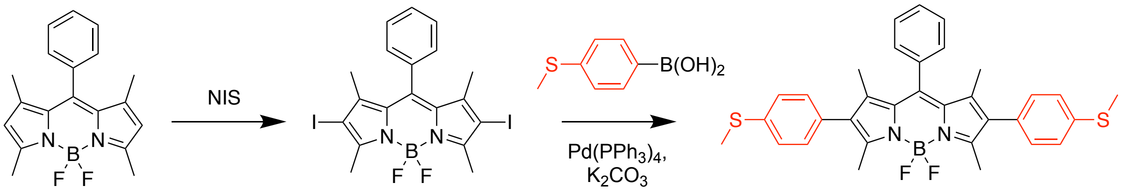

I1

B1

I1. 2,6-Diiodo-8-phenyl-1,3,7,9-tetramethyl-BODIPY was synthesized according to a modified literature procedure.[1,2] 8-Phenyl-1,3,7,9-tetramethyl-BODIPY (166 mg, 0.513 mmol) was dissolved in dichloromethane (DCM, 50 mL) in a 250 mL round-bottom flask equipped with a Teflon-coated stir bar. N-Iodosuccinimide (NIS, 462 mg, 2.05 mmol) was added, and the flask was covered with aluminum foil to exclude light. The reaction mixture was stirred at room temperature under ambient atmosphere for 24 h. The mixture was washed with aqueous sodium thiosulfate (3 × 50 mL) and water (3 × 50 mL), then dried over anhydrous $MgSO_4$. Solvent was removed under reduced pressure to give a red solid (241 mg, 0.418 mmol, 81%). This product was used without further purification.

^{1}H NMR (300 MHz, $CDCl_3$) δ 7.50 – 7.55 (m, 3H), 7.22 – 7.29 (m, 2H*), 2.65 (s, 6H), 1.39 (s, 6H). Chemical shifts match literature values.[2]

B1. Synthesis of **2,6-Bis(4-(methylthio)phenyl)-8-phenyl-1,3,7,9-tetramethyl-BODIPY** was adapted from reported procedures.[3,4] Compound **I1** (241 mg, 0.418 mmol) and 4-(methylthio)phenylboronic acid (316 mg, 1.88 mmol) were added to a 100 mL three-neck flask equipped with a reflux condenser and a Teflon-coated stir bar. The flask was flushed with N_2 on a Schlenk line. Separately, a mixture of dioxane (15 mL), water (1.5 mL), and K_2CO_3 (260 mg, 1.88 mmol) was degassed by sparging with N_2 for 30 minutes, then added to the reaction flask via syringe. Tetrakis(triphenylphosphine)palladium(0) (121 mg, 0.105 mmol) was added, and the mixture was heated at 100 °C under reflux for 72 h. After cooling, the solvent was removed under reduced pressure. The residue was dissolved in DCM, washed with water (3 × 50 mL), dried over anhydrous $MgSO_4$, and concentrated under reduced pressure. The crude product was purified by column chromatography using 33% hexanes in DCM as the eluent to afford the product as a red solid. (54 mg, .095 mmol, 23%).

^{1}H NMR (800 MHz, $CDCl_3$) δ 7.51 – 7.46 (m, 3H), 7.34 (d, J = 7.1 Hz, 2H), 7.27 (d, J = 7.8 Hz, 4H), 7.08 (d, J = 8.0 Hz, 4H), 2.53 (s, 6H), 2.50 (s, 6H), 1.30 (s, 6H). ^{13}C NMR (201 MHz, $CDCl_3$) δ 154.23, 142.03, 139.17, 137.28, 135.34, 133.12, 131.30, 130.54, 130.31, 129.24, 129.06, 127.99, 126.28, 15.68, 13.37, 12.71. HRMS (HESI) m/z $[M + H]^+$ calculated for $C_{33}H_{32}BF_2N_2S_2^+$: 568.2099; found: 568.2095.

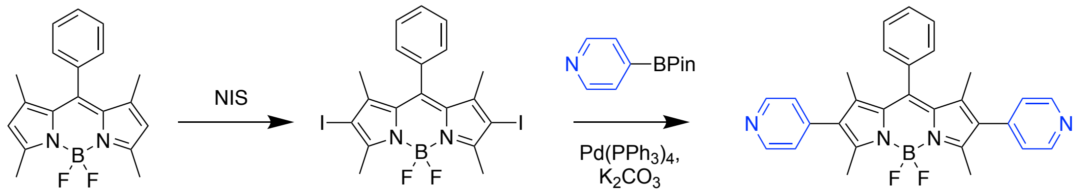

B3

B3. **2,6-Bis(4-pyridyl)-8-phenyl-1,3,7,9-tetramethyl-BODIPY** was synthesized using a modified literature procedure.[3] Compound **I1** was synthesized as described above and combined (148 mg, 0.257 mmol) with 4-pyridylboronic acid pinacol ester (133 mg, 0.649 mmol), and K_2CO_3 (276 mg, 2 mmol) in a 100 mL three-neck flask equipped with a Teflon-coated stir bar. The flask was flushed with N_2 on a Schlenk line. A degassed mixture of dioxane (16 mL) and water (2 mL) was added via syringe, and the solution was sparged with N_2 for 30 minutes. Tetrakis(triphenylphosphine)palladium(0) (22 mg, 0.026 mmol) was added, and the mixture was heated at 95 °C under reflux for 18 h. After cooling, the solvent was removed under reduced pressure, and the residue was dissolved in DCM and washed with water (2 × 50 mL) and brine (1 × 50 mL). The organic layer was dried over anhydrous Na_2SO_4, filtered, and concentrated under reduced pressure. The crude product was purified by column chromatography on triethylamine-treated silica gel using 50% ethyl acetate in hexanes with 1% triethylamine (Et_3N) buffer as the eluent to afford the product as a red solid (63 mg, 0.13 mmol, 51%).

^{1}H NMR (800 MHz, $CDCl_3$) δ 8.64 (d, J = 5.5 Hz, 4H), 7.56 – 7.50 (m, 3H), 7.34 (d, J = 6.9 Hz, 2H), 7.16 (d, J = 5.7 Hz, 4H), 2.57 (s, 6H), 1.35 (s, 6H). ^{13}C NMR (201 MHz, $CDCl_3$) δ 154.30, 148.87, 143.44, 142.80, 140.08, 134.61, 131.68, 130.88, 129.55, 127.67, 125.15, 13.39, 12.75. HRMS (HESI) m/z $[M + H]^+$ calculated for $C_{29}H_{26}BF_2N_4^+$: 478.2249; found: 478.2253. ^{1}H and ^{13}C NMR spectra match literature values.[3]

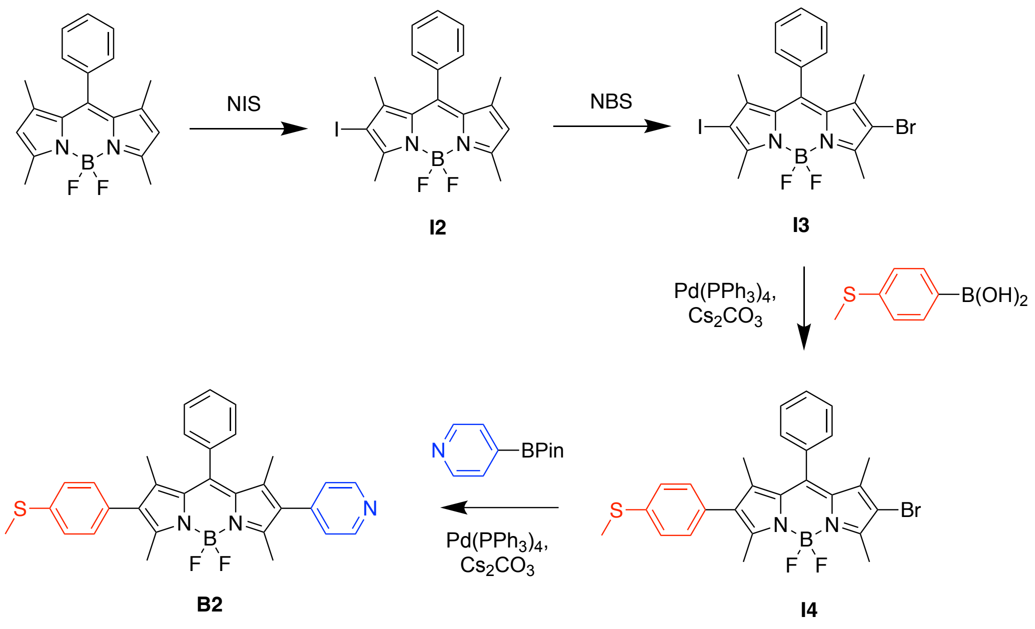

I2. 2-Iodo-8-phenyl-1,3,7,9-tetramethyl-BODIPY. 8-Phenyl-1,3,7,9-tetramethyl-BODIPY (324 mg, 1.00 mmol) was dissolved in DCM (40 mL) in a 100 mL round-bottom flask equipped with a Teflon-coated stir bar. N-Iodosuccinimide (NIS, 225 mg, 1.00 mmol) was added slowly, and the flask was covered with aluminum foil to exclude light. The reaction mixture was stirred at room temperature under ambient atmosphere for 24 h. The resulting mixture was washed with aqueous sodium thiosulfate (1 × 50 mL), water (2 × 50 mL) and brine (1 × 50 mL), then dried over anhydrous Na_2SO_4. The solvent was removed under reduced pressure, and the crude product was purified by column chromatography using 80% hexanes and 20% DCM as the eluent to give the product as an orange solid (310 mg, 0.69 mmol, 69%).

^1H NMR (300 MHz, $CDCl_3$) δ 7.59 – 7.43 (m, 3H), 7.33 – 7.20 (m, 2H*), 6.04 (s, 1H), 2.63 (s, 3H), 2.57 (s, 3H), 1.38 (s, 6H). Chemical shifts match literature values.[5]

I3. 2-Iodo-6-bromo-8-phenyl-1,3,7,9-tetramethyl-BODIPY. In a 100 mL round-bottom flask equipped with a Teflon-coated stir bar, compound **I2** (272 mg, 0.604 mmol) was dissolved in 25 mL of DCM. N-Bromosuccinimide (267 mg, 1.50 mmol) was added, and the flask was covered with aluminum foil to exclude light. The reaction mixture was stirred at room temperature overnight under ambient atmosphere. The mixture was then washed with aqueous sodium thiosulfate (2 × 50 mL), followed by water (2 × 50 mL) and brine (1 × 50 mL), and dried over anhydrous Na_2SO_4. The solvent was removed under reduced pressure, and the crude product was purified by column chromatography using 90% hexanes and 10% DCM as the eluent to give the product as a red solid (284 mg, 0.54 mmol, 89%).

^1H NMR (300 MHz, $CDCl_3$) δ 7.56 – 7.47 (m, 3H), 7.30 – 7.21 (m, 2H*), 2.65 (s, 3H), 2.61 (s, 3H), 1.39 (s, 3H), 1.36 (s, 3H). ^{13}C NMR (126 MHz, $CDCl_3$) δ 156.94, 153.84, 145.32, 141.76, 140.71, 134.58, 131.50, 130.23, 129.55, 129.46, 127.80, 111.88, 85.57, 16.92, 16.03, 13.69.

I4. 2-(4-(Methylthio)phenyl)-6-bromo-8-phenyl-1,3,7,9-tetramethyl-BODIPY. In a 100 mL three-neck flask equipped with a reflux condenser and a Teflon-coated stir bar, compound **I3** (159 mg, 0.302 mmol) was combined with 4-(methylthio)phenylboronic acid (50 mg, 0.296 mmol). The flask was flushed with N_2 on a Schlenk line. Separately, a mixture of dioxane (15 mL), water (2 mL), and K_2CO_3 (200 mg, 1.45 mmol) was sparged with N_2 for 30 minutes, then added to the flask via syringe. Tetrakis(triphenylphosphine)palladium(0) (10 mg, 0.009 mmol) was added, and the mixture was refluxed at 95°C for 24 h. After cooling, the solvent was removed under reduced pressure. The residue was redissolved in DCM, washed with water (2 × 50 mL) and brine (1 × 50 mL), and dried over anhydrous Na_2SO_4. The solvent was removed, and the crude product was purified by column chromatography using 70% hexanes and 30% DCM as the eluent to afford the product as a red solid (110 mg, 0.209 mmol, 72%).

^{1}H NMR (300 MHz, $CDCl_3$) δ 7.54 – 7.48 (m, 3H), 7.33 – 7.24 (m, 4H*), 7.09-7.02 (m, 2H), 2.62 (s, 3H), 2.52 (s, 3H), 2.50 (s, 3H), 1.37 (s, 3H), 1.29 (s, 3H). ^{13}C NMR (126 MHz, $CDCl_3$) δ 156.60, 151.76, 142.01, 140.78, 138.92, 137.73, 134.88, 133.98, 131.64, 130.45, 130.13, 129.80, 129.35, 129.32, 127.91, 126.31, 110.89, 15.63, 13.55, 13.53, 13.49, 12.86.

B2. 2-(4-Pyridyl)-6-(4-(methylthio)phenyl)-8-phenyl-1,3,7,9-tetramethyl-BODIPY. In a 50 mL three-neck flask equipped with a reflux condenser and a Teflon-coated stir bar, compound **I4** (88 mg, 0.17 mmol) and 4-pyridylboronic acid pinacol ester (36 mg, 0.17 mmol) were combined. The flask was flushed with N_2 on a Schlenk line. Separately, a mixture of dioxane (9 mL), water (1 mL), and Cs_2CO_3 (221 mg, 0.68 mmol) was sparged with N_2 for 30 minutes and added to the reaction flask via syringe. Tetrakis(triphenylphosphine)palladium(0) (7 mg, 0.006 mmol) was then added. The reaction mixture was refluxed at 95°C for 24 hours. After cooling, the solvent was removed under reduced pressure. The residue was redissolved in DCM, washed with water (2 × 50 mL) and brine (1 × 50 mL), and dried over anhydrous Na_2SO_4. The crude product was purified by column chromatography on triethylamine-treated silica using 25% ethyl acetate in hexanes with 1% Et_3N buffer as the eluent, affording the product as a red solid (63 mg, 0.12 mmol, 72%).

^{1}H NMR (500 MHz, $CDCl_3$) δ 8.62 (d, J = 6.1 Hz, 2H), 7.55 – 7.45 (m, 3H), 7.35 (dd, J = 7.7, 1.7 Hz, 2H), 7.27 (d, J = 8.5 Hz, 2H), 7.14 – 7.04 (m, 4H), 2.56 (s, 3H), 2.55 (s, 3H), 2.50 (s, 3H), 1.32 (s, 3H), 1.31 (s, 3H). ^{13}C NMR (126 MHz, $CDCl_3$) δ 156.30, 152.22, 149.74, 142.56, 142.24, 140.40, 138.55, 137.67, 135.06, 133.99, 131.94, 131.01, 130.45, 130.33, 129.85, 129.36, 129.25, 127.89, 126.29, 125.04, 15.61, 13.53, 13.20, 12.83, 12.58. HRMS (HESI) m/z $[M + H]^+$ calculated for $C_{31}H_{29}BF_2N_3S^+$: 523.2174; found: 523.2180.

*Peaks marked with an asterisk are partially overlapped by the residual $CHCl_3$ peak. Reported integrations are consistent with expected values.

2. Conductance Measurements

Single-molecule junction experiments were performed using the STM-BJ technique, as previously reported.[6,7] For each compound, a 100 μM solution in 1,2,4-trichlorobenzene is prepared. A few drops of the solution are added to a gold-coated steel substrate. A 0.25 mm diameter Au tip is brought into and out of contact with the substrate in the presence of the target molecule. While the tip and substrate continuously form and break contact, a voltage bias (V) is applied with a 100kΩ in series and the current across the junction (I) is monitored. Conductance (G = I/V) is thus determined.

For each molecule, we collect 5,000 consecutive conductance traces (plotted as conductance vs. tip displacement) and use these without data selection to construct histograms that yield statistically meaningful conductance values. One-dimensional histograms are built using logarithmic bins (100/decade) along the conductance axis. Two-dimensional histograms use logarithmic bins (100/decade) for conductance and linear bins (1000/nm) for tip displacement. Data collection and analysis were performed using custom software written in Igor Pro (Wavemetrics).

Bias dependence measurements were carried out on the same instrument with a modified protocol. For each trace, the tip movement was paused at a fixed time following the start of the measurement. While the tip remained stationary the voltage was ramped from 0 to 1.5 V and back to 0 V, while current was recorded. Before the ramp, the junction was briefly held at a constant bias of 500 mV. Traces exhibiting a stable Au–molecule–Au junction during this initial hold period were identified based on the measured conductance and selected for further analysis. Since the voltage ramp is at a fixed time during the trace, we do not always trap a molecule during this period. The yield for junction formation during the voltage ramp is between 10-20%. These selected traces were used to generate two-dimensional current-time histograms, as shown in Figure 3 of the manuscript.

3. Density Functional Theory Calculations

All the DFT calculations were carried out using density functional theory with FHI-aims software.[8] For each molecule, the geometry was first optimized for an isolated system. A second relaxation was performed after adding a single Au atom to each linker as an Au-N or Au-S bond. Au pyramids (model electrodes) were then appended to each Au atom along the axis of the optimized Au-N or Au-S bond, and this geometry was used in the subsequent transmission calculation. A non-empirical generalized gradient-corrected approximation (Perdew-Burke-Ernzerhof, PBE) for the exchange-correlation functional was used.[9] Scalar relativistic corrections to the kinetic energy were incorporated into the first-principles calculations at the atomic zeroth-order regular approximation (ZORA) level.[10] The Kohn-Sham states were represented in an optimized all-electron numeric atom-centered basis set with "tight" computational settings (roughly equivalent to "double zeta + polarization" quality for all atoms). The calculation results were obtained using standard convergence criteria in the self-consistent field cycle for the difference in the particle density (10^{-5} electrons/Å^3), total energy (10^{-6} eV), sum for Kohn-Sham eigenvalues (10^{-4} eV) and forces (5×10^{-3} eV/Å). The energy-dependent transmission functions were calculated using the non-equilibrium Green's function formalism with the transport package AITRANSS.[11–13] The junction electrodes were modeled by tetrahedral clusters each consisting of 58 gold atoms.

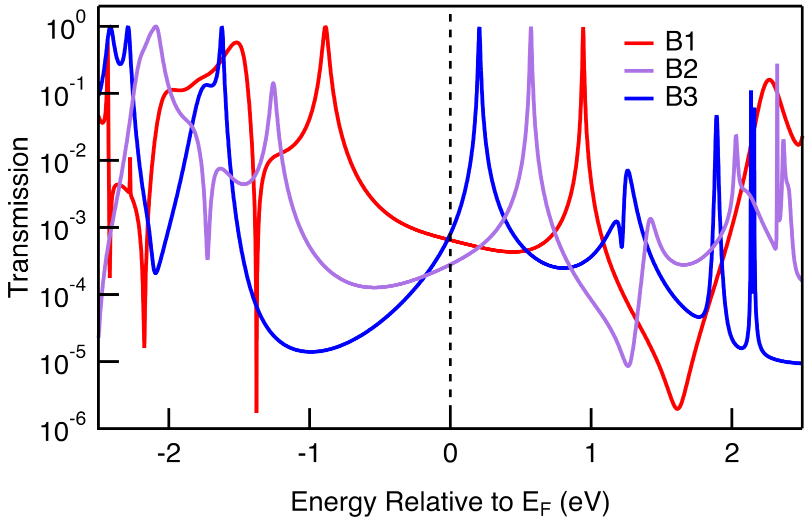

Figure S1. Transmission functions calculated using FHI-Aims and AITRANSS using PBE function for all three molecular junctions.

For the isolated molecule orbital energy calculations, we simply minimized the energy of the molecule using both PBE and B3LYP functionals. The energies of the 8 orbitals closest to the junction Fermi energy were used to correct the transmission functions as explained in the main text.

		PBE-derived energy (eV)	B3LYP-derived energy (eV)	Shift (eV)
B3	LUMO+3	-1.89291	-1.13127	0.76164
	LUMO+2	-2.01350	-1.23391	0.77959
	LUMO+1	-2.04037	-1.25435	0.78602
	LUMO	-3.53115	-2.99495	0.53620
	HOMO	-5.37883	-5.93249	-0.55366
	HOMO-1	-5.97500	-6.89847	-0.92347
	HOMO-2	-5.97644	-7.04685	-1.07041
	HOMO-3	-6.05058	-7.24716	-1.19658
B2	LUMO+3	-1.74776	-0.99275	0.75501
	LUMO+2	-1.78995	-1.03599	0.75396
	LUMO+1	-1.89969	-1.12853	0.77116
	LUMO	-3.32653	-2.80746	0.51907
	HOMO	-5.00368	-5.65865	-0.65497
	HOMO-1	-5.31147	-5.99392	-0.68245
	HOMO-2	-5.87169	-6.77715	-0.90546
	HOMO-3	-5.91394	-7.00242	-1.08848
B1	LUMO+3	-1.39772	-0.65600	0.74172
	LUMO+2	-1.62351	-0.87954	0.74397
	LUMO+1	-1.66468	-0.92324	0.74144
	LUMO	-3.14240	-2.63968	0.50272
	HOMO	-4.80465	-5.45770	-0.65305
	HOMO-1	-5.02991	-5.80463	-0.77472
	HOMO-2	-5.21777	-5.93111	-0.71334
	HOMO-3	-5.83968	-6.72460	-0.88492

Table S1. Calculated energy shifts between PBE-derived and B3LYP-derived isolated molecular orbital energies.

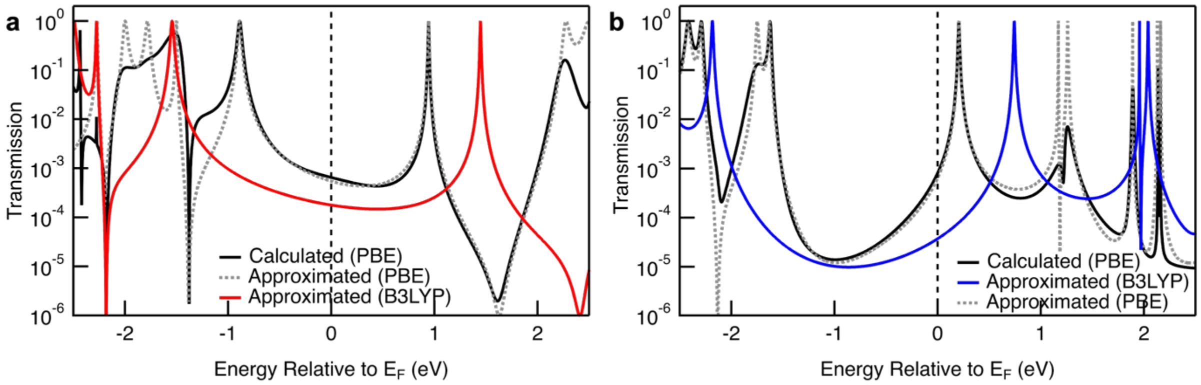

Figure S2. Correction method for PBE transmission as detailed in the manuscript for (a) **B1** junctions and (b) **B3** junctions.

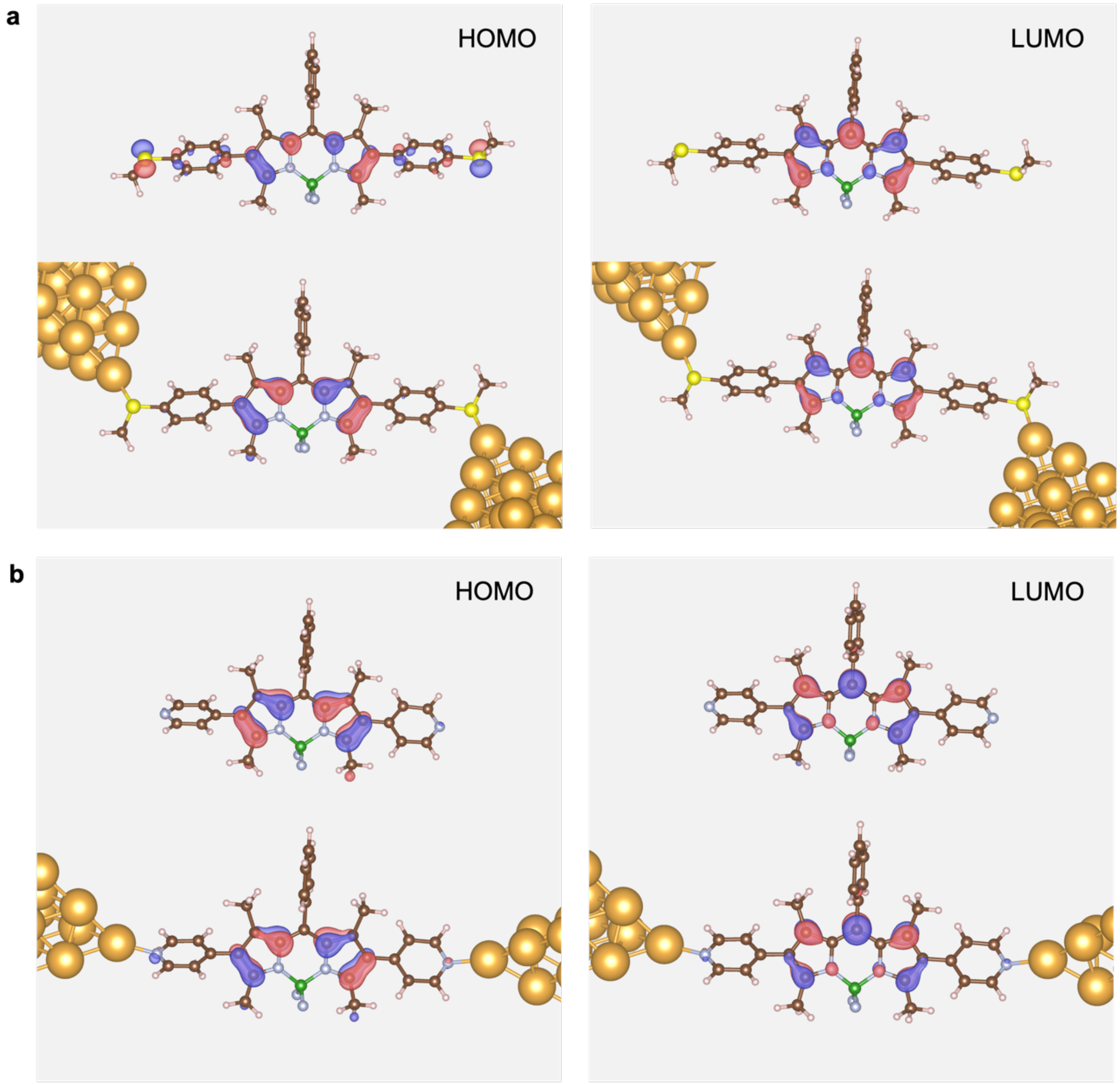

Figure S3. Isosurface plots of (a) **B1** and (b) **B3** frontier orbitals for isolated geometries and for the corresponding electrode-bound systems.

4. NMR Spectra

^{1}H-NMR of **B1** or **2,6-Bis(4-(methylthio)phenyl)-8-phenyl-1,3,7,9-tetramethyl-5,5-difluoro-4,4-diaza-5-bora-s-indacene** (800 MHz, $CDCl_3$, 25°C)

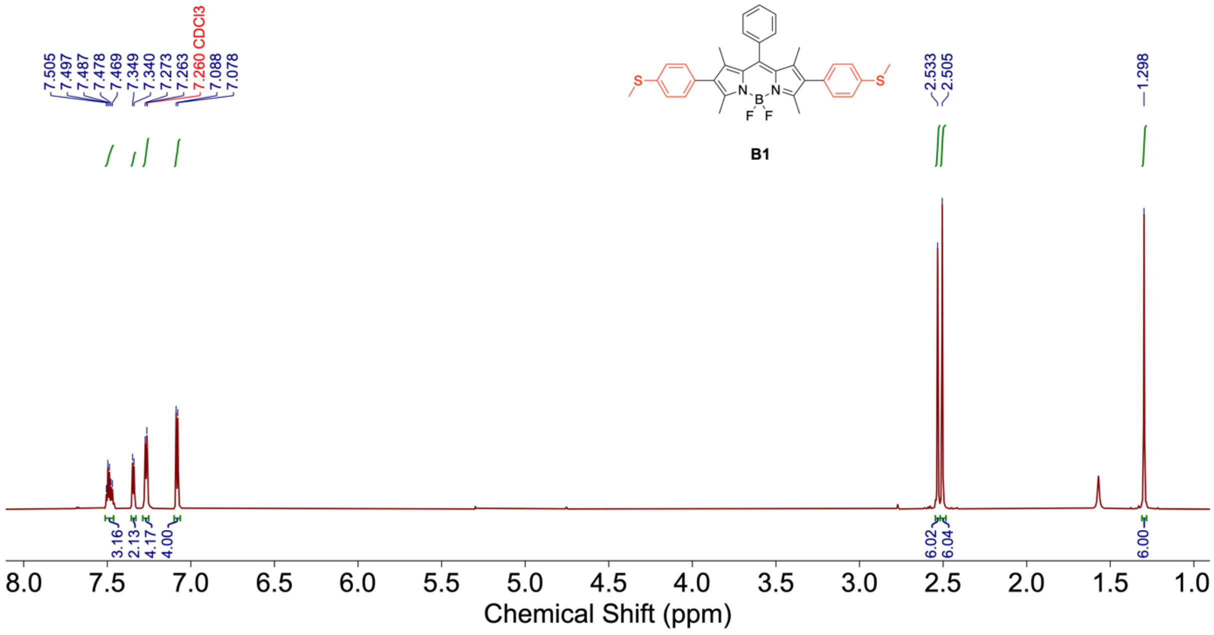

^{13}C-NMR of **B1** or **2,6-Bis(4-(methylthio)phenyl)-8-phenyl-1,3,7,9-tetramethyl-5,5-difluoro-4,4-diaza-5-bora-s-indacene** (201 MHz, $CDCl_3$, 25°C)

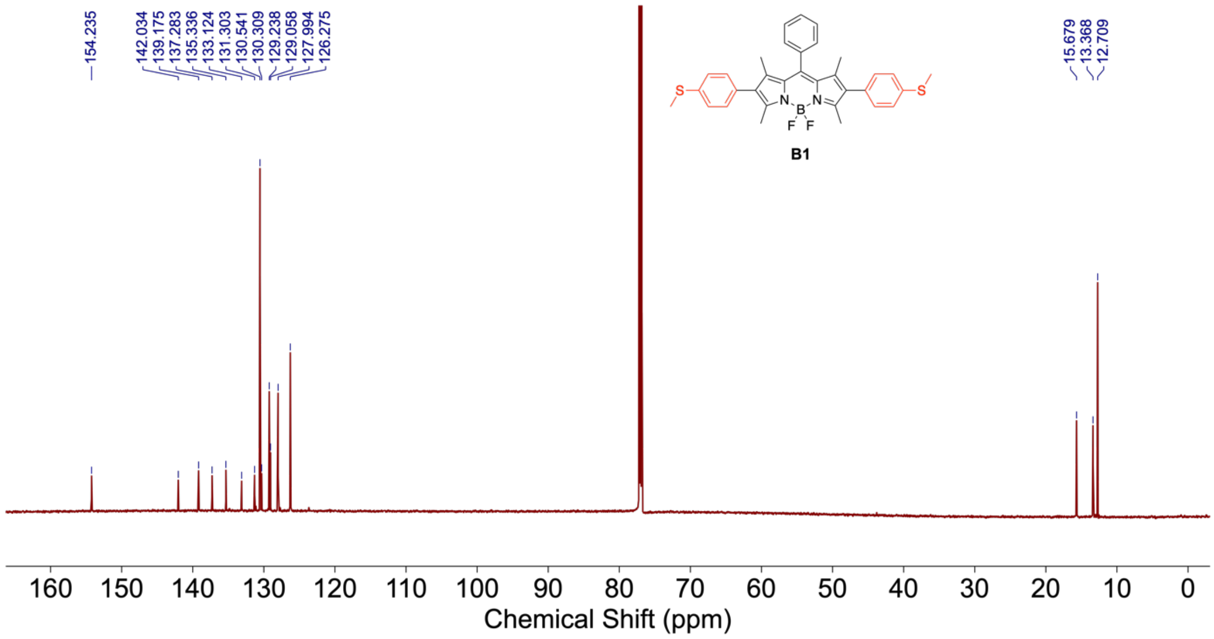

^{1}H-NMR of **B3** or **2,6-Bis(4-pyridyl)-8-phenyl-1,3,7,9-tetramethyl-5,5-difluoro-4,4-diaza-5-bora-s-indacene** (800 MHz, $CDCl_3$, 25°C)

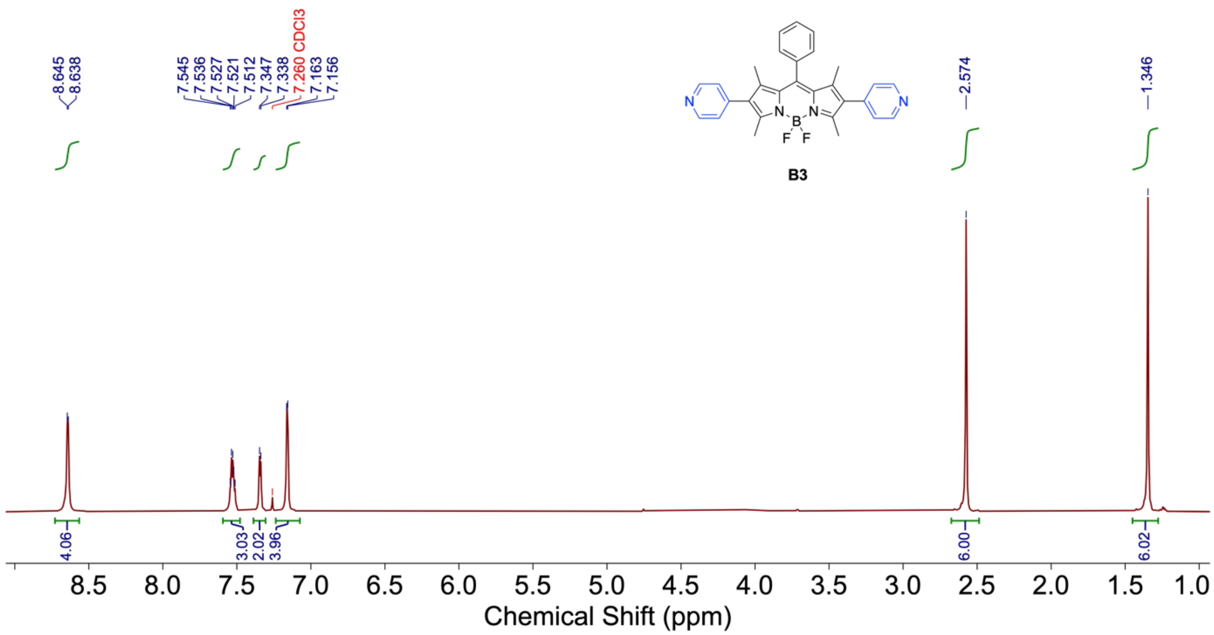

^{13}C-NMR of **B3** or **2,6-Bis(4-pyridyl)-8-phenyl-1,3,7,9-tetramethyl-5,5-difluoro-4,4-diaza-5-bora-s-indacene** (201 MHz, $CDCl_3$, 25°C)

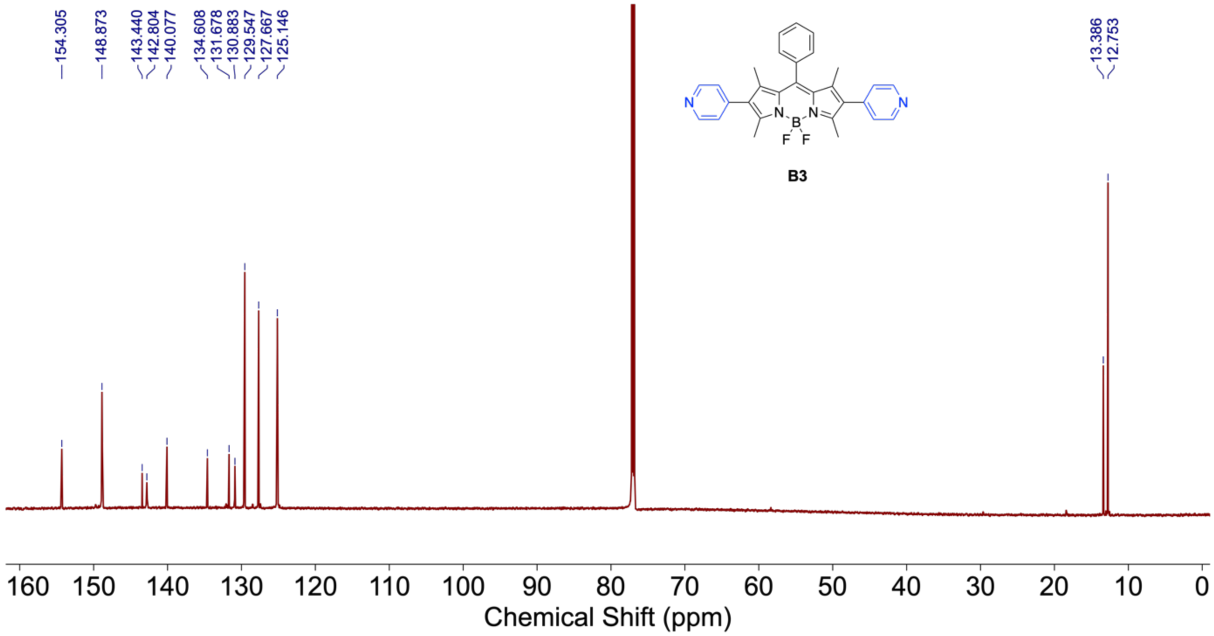

^{1}H-NMR of **B2** or **2-(4-pyridyl)-6-(4-(methylthio)phenyl)-8-phenyl-1,3,7,9-tetramethyl-5,5-difluoro-4,4-diaza-5-bora-s-indacene** (500 MHz, $CDCl_3$, 25°C)

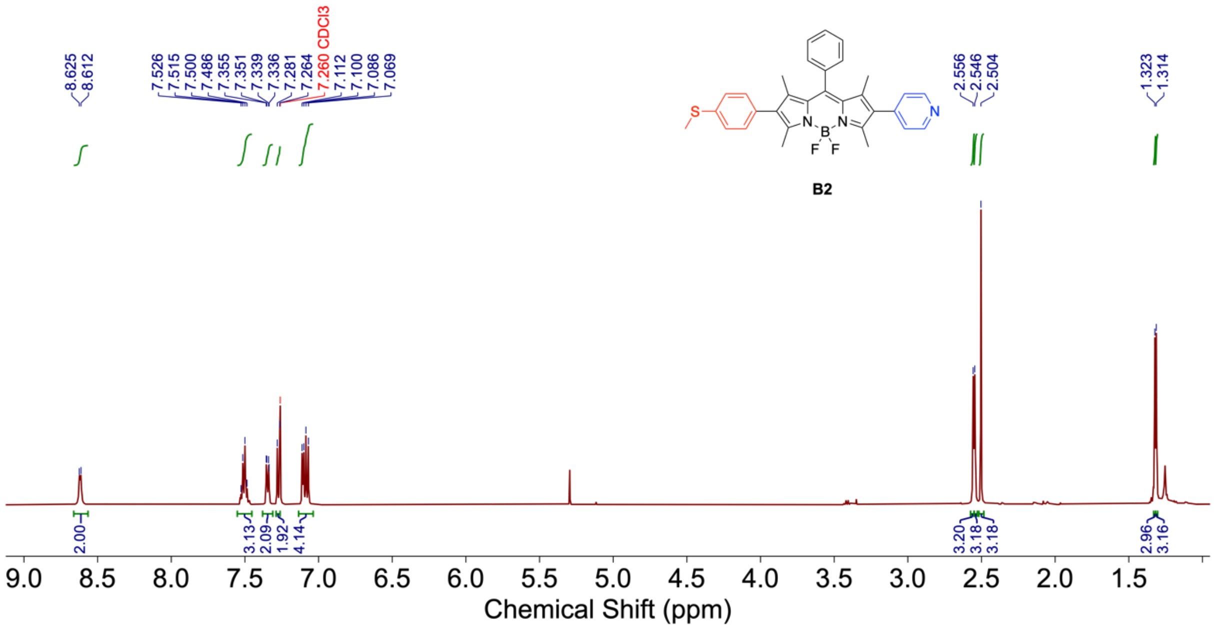

^{13}C-NMR of **B2** or **2-(4-pyridyl)-6-(4-(methylthio)phenyl)-8-phenyl-1,3,7,9-tetramethyl-5,5-difluoro-4,4-diaza-5-bora-s-indacene** (126 MHz, $CDCl_3$, 25°C)

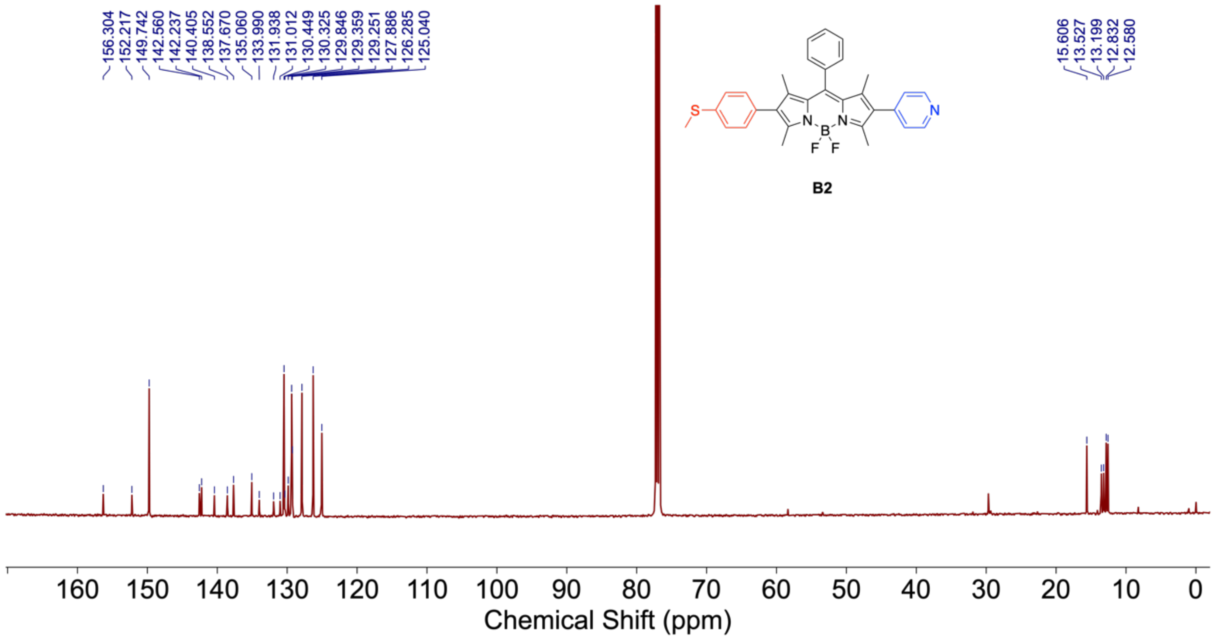

5. References

(1) Ulrich, G.; Ziessel R Fau - Harriman, A.; Harriman, A. The Chemistry of Fluorescent Bodipy Dyes: Versatility Unsurpassed. No. 1521-3773 (Electronic).

(2) Zhang, D.; Wen, Y.; Xiao, Y.; Yu, G.; Liu, Y.; Qian, X. Bulky 4-Tritylphenylethynyl Substituted Boradiazaindacene: Pure Red Emission, Relatively Large Stokes Shift and Inhibition of Self-Quenching. *Chem. Commun.* **2008**, No. 39, 4777–4779. https://doi.org/10.1039/B808681H.

(3) Gupta, G.; Das, A.; Park, K. C.; Tron, A.; Kim, H.; Mun, J.; Mandal, N.; Chi, K.-W.; Lee, C. Y. Self-Assembled Novel BODIPY-Based Palladium Supramolecules and Their Cellular Localization. *Inorganic Chemistry* **2017**, *56* (8), 4615–4621. https://doi.org/10.1021/acs.inorgchem.7b00260.

(4) Mao, M.; Li, Q.-S.; Zhang, X.-L.; Wu, G.-H.; Dai, C.-G.; Ding, Y.; Dai, S.-Y.; Song, Q.-H. Effects of Donors of Bodipy Dyes on the Performance of Dye-Sensitized Solar Cells. *Dyes and Pigments* **2017**, *141*, 148–160. https://doi.org/10.1016/j.dyepig.2017.02.017.

(5) Wu, W.; Guo, H.; Wu, W.; Ji, S.; Zhao, J. Organic Triplet Sensitizer Library Derived from a Single Chromophore (BODIPY) with Long-Lived Triplet Excited State for Triplet–Triplet Annihilation Based Upconversion. *J. Org. Chem.* **2011**, *76* (17), 7056–7064. https://doi.org/10.1021/jo200990y.

(6) Xu, B.; Tao, N. J. Measurement of Single-Molecule Resistance by Repeated Formation of Molecular Junctions. *Science* **2003**, *301* (5637), 1221–1223. https://doi.org/10.1126/science.1087481.

(7) Venkataraman, L.; Klare, J. E.; Tam, I. W.; Nuckolls, C.; Hybertsen, M. S.; Steigerwald, M. L. Single-Molecule Circuits with Well-Defined Molecular Conductance. *Nano Letters* **2006**, *6* (3), 458–462. https://doi.org/10.1021/nl052373+.

(8) Blum, V.; Gehrke, R.; Hanke, F.; Havu, P.; Havu, V.; Ren, X.; Reuter, K.; Scheffler, M. Ab Initio Molecular Simulations with Numeric Atom-Centered Orbitals. *Computer Physics Communications* **2009**, *180* (11), 2175–2196. https://doi.org/10.1016/j.cpc.2009.06.022.

(9) Perdew, J. P.; Burke, K.; Ernzerhof, M. Generalized Gradient Approximation Made Simple. *Physical Review Letters* **1996**, *77* (18), 3865–3868. https://doi.org/10.1103/PhysRevLett.77.3865.

(10) Lenthe, E. van; Baerends, E. J.; Snijders, J. G. Relativistic Regular Two-component Hamiltonians. *The Journal of Chemical Physics* **1993**, *99* (6), 4597–4610. https://doi.org/10.1063/1.466059.

(11) Arnold, A.; Weigend, F.; Evers, F. Quantum Chemistry Calculations for Molecules Coupled to Reservoirs: Formalism, Implementation, and Application to Benzenedithiol. *The Journal of Chemical Physics* **2007**, *126* (17), 174101. https://doi.org/10.1063/1.2716664.

(12) Bagrets, A. Spin-Polarized Electron Transport Across Metal–Organic Molecules: A Density Functional Theory Approach. *Journal of Chemical Theory and Computation* **2013**, *9* (6), 2801–2815. https://doi.org/10.1021/ct4000263.

(13) Wilhelm, J.; Walz, M.; Stendel, M.; Bagrets, A.; Evers, F. Ab Initio Simulations of Scanning-Tunneling-Microscope Images with Embedding Techniques and Application to C58-Dimers on Au(111). *Physical Chemistry Chemical Physics* **2013**, *15* (18), 6684–6690. https://doi.org/10.1039/C3CP44286A.
